# The Health Systems Funding Platform: Is this where we thought we were going?

**DOI:** 10.1186/1744-8603-7-16

**Published:** 2011-05-19

**Authors:** Peter S Hill, Peter Vermeiren, Katabaro Miti, Gorik Ooms, Wim Van Damme

**Affiliations:** 1 Australian Centre for International and Tropical Health School of Population Health The University of Queensland Brisbane, Queensland, Australia; 2 Department of Public Health Institute of Tropical Medicine, Antwerp, Belgium; 3 Department of Political Science University of Pretoria, Pretoria, South africa

## Abstract

**Background:**

In March 2009, the Task Force for Innovative International Financing for Health Systems recommended "a health systems funding platform for the Global Fund, GAVI Alliance, the World Bank and others to coordinate, mobilize, streamline and channel the flow of existing and new international resources to support national health strategies." Momentum to establish the Health Systems Funding Platform was swift, with the World Bank convening a Technical Workshop on Health Systems Strengthening (HSS), and serial meetings organized to progress the agenda. Despite its potential significance, there has been little comment in peer-reviewed literature, though some disquiet in the international development community around the scope of the Platform and the capacity of the partners, which appears disproportionate to the available information.

**Methods:**

This case study uses documentary analysis, participant observation and 24 in-depth interviews to examine the processes of development and key issues raised by the Platform.

**Results:**

The findings show a fluid and volatile process, with debate over whether ongoing engagement in HSS by Global Fund and GAVI represents a dilution of organizational focus, risking ongoing support, or a paradigm shift that facilitates the achievement of targeted objectives, builds systems capacity, and will attract additional resources. Uncertainty in the development of the Platform reflects the flexibility of the recently formed global health initiatives, and the instability of donor commitments, particularly in the current financial climate. But implicit in the conflict is tension between key global stakeholders over defining and ownership of the health systems agenda.

**Conclusions:**

The tensions appear to have been resolved through a focus on national planning, applying International Health Partnership principles, though the global financial crisis and key personnel changes may yet alter outcomes. Despite its dynamic evolution, the Platform may offer an incremental path towards increasing integration around health systems, that has not been previously possible.

## Background

On 10 March 2009, Julian Lob-Levyt, (then) Chief Executive Officer of the GAVI Alliance (GAVI), and Michel Kazatchkine, Executive Director of The Global Fund to fight AIDS, Tuberculosis and Malaria (Global Fund), wrote to Gordon Brown, Prime Minister of the United Kingdom and Robert Zoellick, President of the World Bank (WB), co-chairs of the Taskforce on Innovative International Financing for Health Systems. Their letter presented an ambitious challenge: "It is time to take a comprehensive approach with the necessary support from key donors to refocus on all of the health-related MDGs as a renewed commitment to meeting the basic health service delivery needs in poor countries. We are willing and keen to do this." [1] The response was swift: within a week, the Task Force meeting had formulated a recommendation to "Establish a health systems funding platform for the Global Fund, GAVI Alliance, the World Bank and others to coordinate, mobilize, streamline and channel the flow of existing and new international resources to support national health strategies." [2] In June 2009, the WB hosted a Technical Workshop on Health Systems Strengthening as a first response to the Task Force's call,[3] exploring Health Systems Strengthening (HSS) with a view to informing the development of the proposed funding platform.

The shift towards health systems strengthening is one of the more recent trends in a decade of substantial change, as globalization has increasingly impacted on health and development. The turn of the millennium brought with it a raft of new initiatives: broad agreement on the Millennium Development Goals refocused development towards poverty reduction, including specific health goals for maternal and child health, AIDS, malaria and tuberculosis [4]. New initiatives offered innovative partnerships between public and private sectors, specifically addressing the MDGs and other strategies, with new sources of funding from foundations and philanthropic organizations and an explosion of new structures and relationships [5]. Multiple new players, in complex networks of policy influence, have challenged the constraints of existing aid architecture, demanding new understandings of global governance [6]. With substantial progress in targeted programs, there has been increasing recognition of the need to support health systems more broadly, with the proposal for a joint funding platform signaling, for many, a significant shift in global policy direction [7].

Given this dynamic context, for an initiative of such potential moment, subsequent debate in the literature has been parsimonious. Following the Technical Workshop in Washington, Ruth Levine's breezy blog [8] pictured a "three-way marriage" for GAVI, Global Fund and WB that "brings in the World Health Organization as the maid of honor", arguing for a more open process with greater consultation, particularly with potential recipients of the funding. Despite an initial relative silence, GAVI and WB websites [9,10] are recently carrying more comprehensive notes of the process to date. But in the peer-reviewed literature, reference to the joint platform has been limited, and largely indirect: questions based on current GAVI and Global Fund roles and structures and the track record of WB in health systems [11]; explorations of GAVI's recent entry into HSS [12-14] and some tangential consideration of the implications of the proposed platform for global governance [15]. The background papers prepared for the Technical Workshop-accessible from the WB website [3], but still marked "not available for citation or quotation"-have only recently begun to translate into the academic literature [16]. But a statement issued by Action for Global Health [17] on behalf of some 25 Civil Society Organizations-including signatories such as the International Planned Parenthood Federation, Oxfam International and World Vision International, as well as several national organizations-suggest levels of anxiety within the development community that seem disproportionate to the available concrete information. Their statement raised concerns around the respective roles of the partners, questioning the assumed leadership of WB in preference to the World Health Organization (WHO), the lack of clarity of the structure and its relationship to other donor architecture, available funding, the need for a commitment to universal access to health services and the level of engagement of civil society.

This research seeks to identify and explore the key issues that have arisen in the development of the Health Systems Funding Platform, examining their implications for current directions in development assistance and global health governance. The case-study recognizes that this is a dynamic process, and that issues that have been raised in the research process are in flux: some issues which have been contentious appear to be resolved; structures and processes that have been proposed continue to evolve; significant new directions may yet emerge. Despite this, the research offers an insight into the contribution of the global health partnerships to global health policy processes, with their flexibility and responsiveness, influential leadership and capacity to engage new paradigms, and their vulnerability to global financial and policy trends, and changing preferences of bilateral donors, and private funding sources.

## Methods

This research uses a case-study design [18] to examine the development of the Health Systems Funding Platform, drawing on discourse analysis to locate the evolution of the Platform within prevailing debates around development and global health governance [19]. In seeking to examine contemporaneous issues, the research faces the challenges of both temporality-tracking change in a fluid process-and positionality, with 'outsider' status offering the advantages of independence but constraints in directly accessing more confidential data [20,21].

In order to enhance its rigor [22] and validity [23,24], the research design triangulates three qualitative research methods to examine how the progress towards increasing engagement in HSS, and in particular, the Health Systems Funding Platform, is constructed by key stakeholders. The first is a review of the available literature referring to these processes, including the peer-reviewed academic literature, reports, media releases and website postings [25]. Secondly, this has been supplemented by participant observation of the authors and their colleagues in processes related to HSS (participation in development of HSS applications, analysis and evaluation of proposals, membership of relevant boards, advisory groups and committees, related research) [26]. The third method involved 24 in-depth interviews, with informants purposively selected to provide insight into the evolution of the global health initiatives and their increasing engagement in HSS [22,27]. Informants were selected from a range of institutional affiliations and roles or functions, and included officers responsible for the development of the Platform in each of its partner organizations. In several cases, institutional affiliations or roles were multiple: the most relevant has been chosen to demonstrate the spread of informants in Table 1, and served as a focus for the interview. Table 2 provides a timeline of the key events in the development of the Platform.

**Table 1:** Interview informant matrix

Role/Function	Institutional Affiliation					
	***Bilateral Donors***	***Multilateral Agencies***	***GAVI Global Fund***	***Academic Institutions***	***Civil Society***	***Country Partners***

Government	X					X

Board Member			X			

Senior Management	X	X	X	X		
		X	X			
		X	X			
		X				

Program Management	X	X		X		X
	X			X		

Policy Analysis				X	X	
				X	X	

Technical Advisors				X		
				X		

**Table 2:** Timeline of key events for the Health Systems Funding Platform

Date	Event
10 March 2009	Letter to Task Force on Innovative International Financing for Health Systems from GAVI Alliance and Global Fund.
13 March 2009	Taskforce on Innovative International Financing for Health Systems Recommendation 9: 'Establish a health systems funding platform...'
25-27 June 2009	Health Systems Strengthening: a Technical Workshop hosted by the World Bank, Global Fund and GAVI Alliance, Washington DC.
October 2009	Action for Global Health. Delivering for Health Systems Strengthening: Civil Society Organisations' Comments on the Proposed Joint Platform for Health Systems Strengthening.
26 March 2010	Work Plan for 2010: Health Systems Funding Platform.
June-July 2010	Nepal and Ethiopia complete JANS processes; MOH Cambodia approves areas of cooperation.
4-5 October 2010	Global Fund 2011-2013 Voluntary Replenishment Meeting
6 October 2010	GAVI Alliance 2010-2015 Replenishment Process
October 2010	Julian Lob-Levyt resigns as CEO, GAVI Alliance
November 2010	Dagfinn Høybråten voted new Board Chair of GAVI Alliance

The interviews were conducted in two clusters from September 2009 to November 2010: the first 13 interviews were conducted by one researcher (PV), and acted as framing interviews, using an open format to explore key issues identified by informants around the recent development of GAVI and the Global Fund, and their links to global governance. The second cluster of 11 semi-structured interviews was conducted by a second researcher (PSH) and used a question guide developed following the analysis of the framing interviews. The second interviews explored in more specificity the engagement of the agencies in HSS and the development of proposals for a joint platform for HSS. Interviews with selected informants have been subsequently updated through email and telephone contact.

All interviews, with one exception, were digitally recorded, and transcribed for analysis. Thematic analysis of the first thirteen interviews was undertaken manually by one researcher (MK), and by a second researcher (PSH) using NVIVO 8 qualitative analysis software program (QSR International); differences in themes were discussed and a consensus reached and the interviews recoded. The agreed themes were subsequently used in the analysis of the second set of interviews, and the resulting analysis structure reviewed by all researchers [27]. The research proposal was approved by the Institutional Review Board of the Institute of Tropical Medicine, Antwerp (Approval Number 100727100).

## Results and Discussion

The presentation of the analysis that follows integrates the findings obtained through the observation, interviews and subsequent updates, together with information available through the scientific literature and web-sites. The researchers are responsible for the analysis and conclusions, and have previously indicated their support for a significantly expanded global funding base for health [28].

### The shift towards Health Systems Strengthening

Launched in 2001, GAVI had emerged from the Children's Vaccine Initiative as a public-private partnership that would optimize access to currently underused vaccines, strengthen health and immunisation systems in countries, and make innovative immunisation technology available-particularly in developing countries [29]. In December 2005 the Board of the GAVI Alliance opened a new 'window' for HSS initiatives, marking a substantial broadening of the scope of its funding [30]. This was a distinct paradigm shift for a partnership barely five years old, created with a specific immunisation-related mandate. The decision to engage HSS was contested within the GAVI Alliance Board, but endorsed by a narrow margin. The Global Fund, launched in 2002 with a focus on the triple threats of HIV/AIDS, tuberculosis and malaria [31], later followed GAVI with a single round of dedicated HSS funding, with similar ambivalence within the Global Fund Board [32]. For GAVI, the engagement with HSS has had qualified success [12-14], in part attributed by informants to its containment at 13% of GAVI funds, and its defined "niche focus on eliminating health system bottlenecks". In contrast, the Global Fund's experiment was described by respondents as "messy" and "inconsistent", with a single round of designated HSS funding, subsequently discontinued and replaced by national plan based funding. Although up to 35% of Global Fund allocations were reported as strengthening health systems, the activities were often diffuse and difficult to define. For advocates of a narrower mandate, this level of health systems allocations was of concern. Those supportive of health systems strengthening questioned the extent to which 'health systems activities' contributed only to targeted programs, rather than strengthening the health system as a whole. Reviews of health workforce issues raised by Global Fund activities marked the tension between addressing its three target diseases and broader health systems impacts [33]. For the Global Fund, the Platform would offer some potential resolution: a way to 'clean up' its untidy engagement in HSS-and redress the earlier critiques of its HSS approach by its own Technical Review Panel in 2005 [34].

The 2009 proposal for a joint platform for HSS has re-evoked these same debates within both Boards. Muraskin [35], in his earlier analysis of GAVI, had identified tensions between those board members for whom the "primacy of immunisation is *non-negotiable*" and those-particularly among the bilateral donors-who advocated for a broader systems approach in which immunisation was "integrated with, and subordinated to, broader systems objectives". These divisions persist: proponents of HSS argued that the global health initiatives have picked the "low hanging fruit", and now need to extend into HSS if they are to achieve their own specific mandates. The GAVI Alliance's own commissioned research had increasingly recommended strategies that articulated with the health system as whole. The Millennium Development Goals (MDGs) were seen as defining the urgency of the overall task, but with the expanded demands of the MDGs also offering substantial additional capital through the International Financing Facility for Immunisation (IFFIm), proposed by the Task Force on Innovative International Financing for Health Systems. Here again, funding pragmatism played a role, with informants arguing that essential donors would only continue to fund GAVI and the Global Fund if they were to extend into HSS; if not, funds were likely to divert to other HSS initiatives. The opposing factions saw the strategy as risking the perception of competence that both global health initiatives have built over the past decade. They argued that "diluting" their mandate through engaging HSS risked their respective "brands", and in a compromised donor market, was gambling with the funds needed for their existing activities. For those who defined the Global Fund as primarily a funding mechanism, with no brief to extend into the broader issues of global health policy, managing its original HSS initiative had already proved problematic. The internal reviews of the experiment provided little reassurance. The current Global Fund replenishment cycle has accentuated these anxieties [36].

But in this debate, as with many policy issues, significant heterogeneity is evident among and within the stakeholders. While the positions of some organizations were caricatured by some informants (Levine's anthropomorphic "marriage" is a humorous example [8]) the diversity and flexibility of positions within organizations was evident in perceptions of most interviewees. Informants remarked on the asymmetry of influence between stakeholders, with potential recipient countries consulted but with their capacity to shape outcomes limited until the proposed models had been articulated. Interestingly, the substantial role of individuals in shaping outcomes was a consistent theme, with the dominance of the same actors identified by both supporters and detractors, the impact of their (then imminent) departures on policy positions weighed. In contrast to the ambivalence of the GAVI Board, its Secretariat appeared unequivocally committed to the joint platform. Its Chief Executive Officer co-signed the original proposal, identified IFFIm funds as a potential source and secured a senior appointment and team to progress the agenda. Leadership within all stakeholder organizations was seen to determine the options available for consideration, with modifications of position anticipated with changes in personnel. Gates himself was reported as explicit in his opposition to the joint platform proposal put to the GAVI Board-a position consistent with his technological focus [37,38]-with his increased personal attendance at Board meetings interpreted as reinforcing his position. The "factional" divisions were not as rigidly polarized as Muraskin [35] would suggest: with three primary partners working on the proposal in separate development processes, and then meeting with WHO to negotiate consensus, stakeholders were provided with multiple points of intervention. As a result of these independent processes, the content of progress reports put to the GAVI board was noted as being different from those put to the Global Fund. More interestingly, key bilateral donors were said to offer differing positions to each board-arising in part from their perceptions of the differing implications of the proposal for the more contained focus of GAVI, and the more polyvalent engagement of Global Fund-and another to the meetings of the WB. Changes within the United States political administration were reflected in their evolving position; perceptions of the appropriateness of applying IFFIm funding to this proposal qualified the position of some bilateral donors, with the Japanese indicating a willingness to contribute directly to the HSS proposal. In particular, WHO's heavy dependence on Global Fund funding for implementation of country HIV/AIDS programs, generated tensions within WHO itself, with factional positions that paralleled the opposing perspectives within the Global Fund Board. The failure to include WHO in the initial conceptualization of the Platform [1] fed into broader agency politics around WB's contested claim over leadership in health systems policy more broadly, and health systems strengthening specifically [39]. WHO's ready acceptance of the role of 'facilitator' for the Platform was seen by some respondents as conceding its designated 'technical' role in health systems strengthening [39]; by others as a pragmatic recognition that, unlike the other three partners, it had limited financial resources of its own to contribute to the Platform.

### Developing the Platform: structure and definition

#### Platform funding

The Task Force for Innovative International Financing for Health Systems met in a climate of economic optimism: in early 2009, the innovative financing mechanisms championed in their report were expected to offer an additional $10 billion a year [2]. Financial expectations for the Platform were initially high: "flash floods of funds" was the description of one informant. Despite the resistance of some stakeholders to the creation of further structures for global health, an environment of ambitious optimism had been created by the resurgence of corporate philanthropy, with its substantial mobilization of development resources through the global health initiatives, and the demonstrable achievements of the AIDS antiretroviral treatment roll-out. Health advocates were arguing for the transformation of the Global Fund into a "Global Health Fund" [40], or together with GAVI Alliance, into a "global fund for the health MDGs" [41]. Not surprisingly the concepts were conflated in the minds of several informants, who spoke of the "global platform" for HSS, and in one case imagined a "global fund for everything". The chastened context of the global financial crisis has changed much of that expectation, the IFFIm funding more modest than anticipated and not unambiguously available to the Platform. Both GAVI and Global Fund informants were quick to put the Platform in perspective, noting the failure of the donor community to embrace proposals for a global fund for health: "It looks like, in the financial barrel at the moment, there's not going to be big up-front, front-loaded resources." As reiterated on GAVI's website: "The Platform is not a global pool of funds: funds will still flow from the participating financers-currently the GAVI Alliance, the Global Fund and the World Bank." [10]

#### Platform governance

Issues of governance-and the impact of the proposed platform on global health governance-were integral to the discourses around the joint platform. The imprimatur of the Task Force on Innovative International Financing for Health Systems had associated the Platform with a global challenge-the health MDGs-and the "pooling of resources at the global level... funds from existing traditional DAH [Development Assistance for Health] and innovative sources" [2]. Concerns around the internal governance of the Platform were coloured by these global connotations, with old fault lines opening up, and deep tensions reactivated. Action for Global Health's position was to "oppose the World Bank adopting a leadership role at global or national level in the proposed joint platform... In contrast to the World Bank, the WHO is the lead UN agency on health and already has the global mandate to lead on health policy and health systems strengthening" [17]. One key informant envisaged the possibility that the Platform had the potential to "define the health systems agenda and the priorities, with substantial funding behind it... a de facto institution... created in parallel to the World Health Assembly but [with] even less sense of accountability than the World Health Assembly itself." In the absence of more nuanced detail for the proposed platform, debate risked devolving to organizational caricatures and threats amplified by historical divisions.

Yet the same simplifications work positively, enabling respondents within the platform partners to conceive of a "collaborative division of labour" [39] with WHO and WB represented as maintaining complementary positions: "It's a coordination of roles, and put simply, for me, the Ministry of Health internationally is WHO... the World Bank is more the Ministry of Finance." In these constructions of the Platform, any anxiety that the lack of a local in-country GAVI or Global Fund presence would automatically lead to WB paradigms dominating the implementation of the Platform were countered by a division of influence: WHO would retain its technical and normative functions; WB its sustainable financing policy and budget frameworks. Characterising their relative strengths had already been used in justifying the partnership, with GAVI's competence in immunisation seen as a logical bridge to a broader MDG4 orbit of responsibility. Shakow's *Global Fund-World Bank HIV/AIDS Programs Comparative Advantage Study *[42] had conceded any lead role in health systems strengthening to the WB, neatly allocating to the Global Fund the strategic financial and technical focus on its three nominated diseases. In an extrapolation of this role division, discussions around the Platform suggested that between the three agencies, the 'health system' could be covered: GAVI extending its coverage to include a broader Maternal and Child Health agenda, the Global Fund adding the neglected tropical diseases and global epidemics to its brief, and WB covering generic finance and systems issues.

Yet this convenient role differentiation has papered over other differences between the partners. Their governance structures differ, with the World Bank's Board accountable to its 187 country members, and in particular, its major contributors. Its structure, rooted in the 1944 Bretton Woods agreements [43], contrasts with the comparable flexibility of the GAVI and Global Fund Boards, with their differing mixes of public and private sector (both industry and civil society) stakeholders. The World Bank's resource allocation processes lack the responsiveness of GAVI and Global Fund processes, and changes to health financing cannot be made without implications for the Bank as a whole. This has constrained WB participation in current models for the Platform. The three hold differing approaches to the sustainability of funding, essential to the long term imagining of the Platform, with the World Bank reluctant to depend on development assistance for permanent increases in recurrent expenditure [44] and Global Fund arguing for sustained international support for recipients [45], but facing the challenges that this brings in its replenishment cycles [36].

Resistance to the joint platform within the boards of both GAVI Alliance and the Global Fund reportedly coalesced around perceptions of "dilution of mandate" and an erosion of focus and efficacy with the broadening of their commitment. Attention was drawn to the distinctive resource allocation processes maintained by the three partners, with some scepticism that WB would be able to adopt the more participatory processes of both GAVI and Global Fund. Yet in the models currently being promoted, the proposal for a common application form for both GAVI and Global Fund has to some extent accommodated this, with WB only engaged in one option of the two tracks proposed, not engaged with GAVI and Global Fund in the harmonisation of existing HSS funding, nor in the common application form process. It will contribute to the total available HSS funding through national planning processes, but will maintain its current evaluation and approval procedures. The adoption of IHP's Joint Assessment of National Strategies (JANS) tool [46] as a basis for funding applications has provided the single most significant mechanism of alignment within the Platform, and with the strong existing linkage of JANS and IHP to the Aid Effectiveness Agenda doing much to neutralise criticisms. Interestingly, in March 2010, while the platform partners, or informants within WHO or IHP itself did not canvas the use of JANS or the platform's alignment with IHP as concrete propositions during interviews, the functions of the platform were consistently conceptualised in terms of the aid effectiveness principles, with one informant speculating that "I know that some people view the platform as the missing part of IHP, where IHP was supposed to come with the promise of money and the platform will be that."

#### Platform scope

Yet beyond the uncertainty around the quantum of funding available, and its implications for governance options, there has been a particular but charged debate around the scope of the proposal: whether it applies to Health Systems or Health Systems Strengthening. The Task Force recommended a "health systems funding platform" [2], though the IFFIm funding is clearly described as a mechanism to "support health systems strengthening" [47]. The World Bank hosted the *Technical Workshop on Health System Strengthening *in June 2009, and a month late a joint meeting was convened in Geneva to discuss the "Joint Programming and Funding Platform for Health System Strengthening" [48]. Action for Global Health's critique is of the proposed "Joint Platform for Health Systems Strengthening" [17], though the eventual formal title seems to have been resolved as the Health Systems Funding Platform [10].

Health systems strengthening continues to be used within documentation in descriptions of the objectives of the platform, though individual informants from both partners and critics of the platform were emphatic that the platform was not a *health systems strengthening *platform, but a *health systems *funding platform, "and that distinction is quite important because there has actually been a whole battle around that". The website of the Technical Workshop appears to allude to that contention:

"The workshop proved very useful in drawing out a diversity of views on Health Systems and Health Systems Strengthening. A couple of points became clear - that while there are a variety of HS frameworks they tend to have common elements; there was little enthusiasm for developing a new "common" HS framework or for more "debate" among frameworks."[3]

Yet this lack of enthusiasm does not imply that consensus around HSS had already been reached. The tensions implicit in the Workshop agenda seem to reflect a deeper conflict over the "territory of health systems strengthening" and by implication, health systems. In part, there was resistance to the "academic agenda" constructed in the Workshop program: the background papers comprehensively mapped the diversity of health systems approaches, and explored potential frameworks for HSS [3]. Yet this exploration itself prompted concern: respondents who had expected the Technical Workshop to focus on the practical mechanics of the platform argued that the program "doesn't talk just about harmonising funding floats, it talks about defining health systems, defining priorities of health systems, defining the frameworks of health systems, defining health systems strengthening". For reasons that are integral to the development of the concepts underlying health systems, the Technical Workshop could not be the locus for that defining to occur.

### Health Systems and the Platform: evolution and resolution

The importance of health systems is not contested. From its World Health Report 2000 [49], WHO has re-iterated its health systems focus in a *Framework for Action *[50], linking it implicitly to the re-launch of its Primary Health Care initiative [51]. Health systems obstacles to achieving the MDGs have been identified [7] with a call for a global focus on health systems reform [52,53]. Evidence of synergy between global health initiatives and health systems has been documented [54] and health systems strengthening re-interpreted through systems theory [55,56]. Yet the scepticism expressed by Marchal et al. [57] around the 'meaning' of health systems strengthening is germane to the defining of the Platform. They argue that global health initiatives (not exclusively GAVI Alliance and the Global Fund), by tying health systems strengthening to their particular mandates, shift from a "comprehensive discourse to a selective practice": on the one hand focussing on specific health systems obstacles to their own narrow objectives; on the other, labelling any capacity-building activity as 'systems strengthening' [57].

In the case of the Health Systems Funding Platform, the tensions around nomenclature can be understood in terms of metonymy, where one element of a concept may be seen to stand for the whole. Beginning from the frame of immunisation (GAVI) or AIDS, Tuberculosis and Malaria (Global Fund), health systems strengthening evokes a health system, but one that is constrained-arguably distorted-by this limited framing. The tension, then, lies between two possible representations of the health system: the system constructed from a HSS premise, differing substantially from the health system framed from a more comprehensive perspective. In short, the Platform had the potential to redefine the health systems agenda as a whole, and not simply the interface with its own composite programs. With the promise of substantial funding, and its structure embracing four dominant development agencies and open to additional partners, the perceived risks were significant. If it was to extend its brief to embrace MDGs 4, 5 and 6, and beyond, as the Task Force had envisaged, reshaping the global development approach to health systems, the Platform needed a different point of departure. In the words of the old joke: "if that's where you want to go, you'd better not be starting from here!"

## Conclusions

This debate, constructed in terms of its threats to global health governance, has been largely resolved by the Platform at the level of the local-the national-health system. The Health Systems Funding Platform has shifted the point of engagement from the global, with its focus on partner relationships, to country level, essentially using IHP+ as the local umbrella for its commitment to one national plan and one financial management framework, harmonizing and aligning their results and monitoring and evaluation plans. The simplification of transaction costs, application and funding mechanisms remains a goal: a common application form will apply for submissions to either GAVI or Global Fund, and countries having completed their Joint Assessment of National Strategies will be able to seek funding in line with their National Health Plan [58], though funding initially will proceed through separate grant agreements. The proposed process takes advantages of structures largely in place, though the pragmatics of changing procedures and synchronising funding cycles in each of the partners mean that in the short term, achieving harmonisation is the goal, rather than complete integration [59]. It does accommodate the necessary variance expected between country applications, and allows for differing local structures (Sector Wide Approaches or other funding pool mechanisms) and alignment with country planning cycles.

The resolution of the structure of the Health Systems Funding Platform speaks both to the flexibility of the global health initiatives, and the volatility of debate around the health systems agenda. Reflecting on the development of the Platform, the elements that comprise the final structure have largely been present at all stages of its evolution-articulated both in the positions of the partners, and of their critics: the recognised need for a health systems approach, the commitment to the Paris Principles and to reduced transaction costs in development planning, the links to the MDGs, the utility of IHP+ engagement with countries. Using Fidler's analogy of open source codes [60], it is as though repackaging of the available elements has become possible, emerging from contested forums-both internal and external-with the reframing of Health Systems stitching the whole concept together [61]. The resultant Platform is neither the threat to global health governance that some had envisaged, nor merely the "modest" procedural alignment posed as a possible outcome by one of the partner informants.

The proposal, as it currently stands, is an elegant but diminished solution to the challenge of maintaining progress towards integration in the face of limited available resources, and the logistics of implementation. It clearly articulates with the progressive movement towards alignment of development assistance and harmonisation of processes, though the initial JANS pilot will include only four to five countries. The engagement with IHP+ is mutually beneficial, making the advantages of the IHP+ principles more concrete to recipients, but providing a structure for engagement and a filter for the Platform by identifying governments committed to collaborative processes. The statement of protest has been withdrawn from the Action for Global Health website. Yet the evidence to date is that the Platform's emergent form continues to be the product of the ongoing interaction with the network of elements to which it is linked: the pool of partners, their leadership, the global economic situation, trends in development assistance and in global health governance. Cumulative changes could cheat it of its potential. The IHP+, to which the Platform's fortunes are shackled, is itself vulnerable, with disquiet around its progress to date and the political linkage through its initial champion, Gordon Brown, now attenuated [62,63]. Julian Lob-Levyt has resigned from his position at the GAVI Alliance, and partners are already negotiating a 'post-Julian' calendar [64]. The failure of the replenishment cycles to meet expectations [36] has re-focused attention on core mandates for GAVI and Global Fund. In reporting that suggests that GAVI "will not expand its remit to engage more fully with revitalising health-care systems", the new chair of its Board, Dagfinn Høybråten is quoted as saying "the economic climate is tight right now so we have to maintain our focus on delivering the vaccines that developing countries are demanding" [65]. Global Fund is reported as wanting to be explicit in its containment of the funding allocation to HSS. World Bank processes have been minimally changed by the Platform. The global financial crisis has already impacted on the commitments of several bilateral donors, and offers no certain future.

But with global health initiatives multiplying and fragmenting to address specific targeted agendas, the Health Systems Funding Platform offers a significant, initial move in the opposite direction, towards synthesis. It may not be the imagined outcome of that ambitious letter to the Taskforce, with its possibility of merging funding streams into a single channel, focusing on health systems at the heart of efforts to achieve MDGs 4, 5 and 6. Yet it constitutes positive progress, a realistic option in the current global political and economic context. Demonstrable results from the first cycle of applications should encourage additional funding: perhaps enough to galvanise a concerted and substantial investment in health systems. The path to the increasing integration of health systems support could prove to be incremental. And we should be able to get there from here.

## Abbreviations

GAVI: GAVI Alliance; Global Fund: The Global Fund to fight AIDS, Tuberculosis and Malaria; HSS: Health Systems Strengthening; IHP+: International Health Partnership Plus; MDGs: Millennium Development Goals; WB: The World Bank; WHO: World Health Organization

## Competing interests

The research was funded by the European Commission through the 'GHIs in Africa' project (INCO-CT-2006-032371). The funders had no influence on the findings of the research. The authors declare no other competing interests.

## Authors' contributions

PSH contributed to the design of the research project, conducted 11 interviews, coded and analyzed the interview data and prepared the first draft of the paper.

PV developed the design of the framing interviews, undertook a literature review and conducted 13 interviews and reviewed the draft paper and revisions.

KM undertook a literature review and coded and analyzed the interview data, and reviewed the draft paper and revisions.

GO consulted on the interviews and reviewed and edited the draft paper and revisions.

WVD contributed to the design of the research project, supervised the preparation of the research ethics approval, and reviewed the draft paper.

All authors have read and approved the final revision of the paper.
